# First-dose analgesic effect of the cyclo-oxygenase-2 selective inhibitor lumiracoxib in osteoarthritis of the knee: a randomized, double-blind, placebo-controlled comparison with celecoxib [NCT00267215]

**DOI:** 10.1186/ar1854

**Published:** 2006-01-16

**Authors:** Ralf H Wittenberg, Ernest Schell, Gerhard Krehan, Roland Maeumbaed, Hans Runge, Peter Schlüter, Taiwo OA Fashola, Helen J Thurston, Klaus J Burger, Ulrich Trechsel

**Affiliations:** 1 Orthopaedische Abteilung, St Elisabeth Hospital, Herten, Germany; 2 Promedica GmbH, Nürnberg, Germany; 3 Offenbachweg 1, Graben-Neudorf, Germany; 4 Praxis Dr Maeumbaed, Höchstadt, Germany; 5 Praxis Dr Runge, Erlangen, Germany; 6 Praxis Dr Schlüter, Hemsbach, Germany; 7 Novartis Pharma AG, Basel, Switzerland; 8 Novartis Pharma GmbH, Nürnberg, Germany

## Abstract

Cyclo-oxygenase-2 selective inhibitors are frequently used to manage osteoarthritis. We compared the analgesic efficacy of the novel cyclo-oxygenase-2 selective inhibitor lumiracoxib (Prexige^®^) versus placebo and celecoxib in patients with knee osteoarthritis. This seven day, double-blind, placebo and active comparator controlled, parallel group study included 364 patients aged ≥50 years with moderate-to-severe symptomatic knee osteoarthritis. Patients received lumiracoxib 400 mg/day (four times the recommended chronic dose in osteoarthritis; *n *= 144), placebo (*n *= 75), or celecoxib 200 mg twice daily (*n *= 145). The primary variable was actual pain intensity difference (100 mm visual–analogue scale) between baseline and the mean of three hour and five hour assessments after the first dose. Actual pain intensity difference, average and worst pain, pain relief and functional status (Western Ontario and McMaster Universities Osteoarthritis Index [WOMAC™]) were measured over seven days. Patients also completed a global evaluation of treatment effect at study end or premature discontinuation. For the primary variable, the superiority of lumiracoxib versus placebo, the noninferiority of lumiracoxib versus celecoxib, and the superiority of lumiracoxib versus celecoxib were assessed by closed test procedure adjusting for multiplicity, thereby maintaining the overall 5% significance level. In addition, celecoxib was assessed versus placebo in a predefined exploratory manner to assess trial sensitivity. Lumiracoxib provided better analgesia than placebo 3–5 hours after the first dose (*P *= 0.004) through to study end. The estimated difference between lumiracoxib and celecoxib 3–5 hours after the first dose was not significant (*P *= 0.185). Celecoxib was not significantly different from placebo in this analysis (*P *= 0.069). At study end 13.9% of lumiracoxib-treated patients reported complete pain relief versus 5.5% and 5.3% of celecoxib and placebo recipients, respectively. WOMAC™ total and subscales improved for both active treatments versus placebo except for difficulty in performing daily activities, for which celecoxib just failed to achieve significance (*P *= 0.056). In the patient's global evaluation of treatment effect, 58.1% of patients receiving lumiracoxib rated treatment as 'excellent' or 'good', versus 48.6% of celecoxib and 25.3% of placebo patients. Lumiracoxib was well tolerated. The overall incidence of adverse events was similar across treatment groups.

## Introduction

Nonsteroidal anti-inflammatory drugs (NSAIDs) are widely regarded as the agents of choice when treating the chronic pain of osteoarthritis (OA) [1-3]. This class of drugs prevents prostaglandin synthesis by nonselectively inhibiting both isoforms of cyclo-oxygenase (COX) [4,5]; this profile also accounts for their common side effects, including gastric irritation, renal impairment and inhibition of platelet aggregation [6-9]. NSAID use is associated with an increased risk for gastrointestinal ulcers and associated ulcer complications such as bleeds and perforations [7]. COX-2 selective inhibitors have demonstrated analgesic and anti-inflammatory efficacies comparable with those of traditional NSAIDs in patients with arthritis, combined with an improved safety profile [10-13].

Lumiracoxib (Prexige^®^, Novartis Pharma AG, Basel, Switzerland) is a novel COX-2 selective inhibitor in development for the treatment of OA and acute pain. Selectivity for COX-2 over COX-1 has been demonstrated for lumiracoxib both *in vitro *and *in vivo *[14]. In addition, lumiracoxib is distinct from other COX-2 selective inhibitors in that it lacks a sulphur-containing moiety but rather possesses a carboxylic acid group, which confers weakly acidic properties (pKa 4.7) [15]. The unique molecular structure translates into a distinct pharmacokinetic profile, such that lumiracoxib has a rapid plasma uptake (T_max _2 hours) and a short mean plasma half-life of approximately 4 hours [16]. The pharmacokinetics of lumiracoxib are characterized by good oral bioavailability [17], dose proportionality with no accumulation, and no significant influence of age, sex, or body weight on apparent plasma clearance [18]. In addition, lumiracoxib has demonstrated sustained higher synovial fluid concentrations compared with plasma concentrations in patients with rheumatoid arthritis [19].

A four-week phase II study evaluated the efficacy of four doses of lumiracoxib (50 mg twice daily, 100 mg twice daily, 200 mg twice daily and 400 mg once daily) in patients with knee or hip OA [20]. All doses reduced OA joint pain intensity, with significance over placebo observed after the first week of treatment.

Rapid onset of analgesia is necessary if patients are to accept traditional NSAIDs or COX-2 selective inhibitor treatment, because these patients often use their medication intermittently on a *pro re nata *(when required) basis [21]. The present study was conducted to evaluate the analgesic efficacy and tolerability of lumiracoxib 400 mg once daily (four times the recommended chronic dose in OA) and to compare them with those of placebo and celecoxib 200 mg twice daily (recommended dose in OA is 200 mg/day, administered as a single dose or as 100 mg twice daily) [22] in patients with OA of the knee over a 7-day period, with particular focus on the onset of analgesia following the first dose.

## Materials and methods

This was a randomized, double-blind, placebo and active comparator controlled, parallel group study conducted in 32 centres in Germany. All patients provided written informed consent before study-related procedures were conducted, and the study was performed in accordance with the principles of good clinical practice and the Declaration of Helsinki (1964 and subsequent revisions). The study consisted of two phases: a single-dose pain assessment phase and a multiple-dose pain assessment phase.

### Patients

Male or female patients aged 50 years or older with moderate-to-severe symptomatic OA of the knee, according to the American College of Rheumatology criteria [23], were eligible for inclusion. At screening, patients were required to be receiving NSAIDs/simple analgesics for their OA and to have pain intensity in the affected target joint of ≥40 mm on a 100 mm visual–analogue scale (VAS) after activity (walking 20 paces on a flat surface).

Exclusion criteria included secondary OA, concomitant significant medical problems, a history of gastrointestinal bleeding, peptic ulceration or open knee surgery within one year of study entry, and hypersensitivity to analgesics, antipyretics, NSAIDs, or sulfonamides. Patients who had undergone observational arthroscopy, arthroscopic surgery, or lavage within the preceding 180 days were not eligible for enrolment. Female patients who were pregnant, lactating, or of childbearing age and not using adequate means of contraception were excluded.

Concomitant treatment with H_2 _receptor blockers, proton pump inhibitors, misoprostol, methotrexate, warfarin, analgesics (other than rescue medication) and systemic corticosteroids was not permitted during the study; neither was physiotherapy for the target joint. Patients who had received intra-articular corticosteroids in the study joint during the three months before the study were excluded. Patients receiving chondroitin and/or glucosamine were excluded. Patients were permitted to use rescue medication (acetaminophen ≤3 g/day) during the study, although use of rescue medication was prohibited from midnight before the baseline clinic visit.

### Study design and treatments

After an initial screening visit patients entered a 2- to 7-day washout period, during which any treatment with NSAIDs/analgesics was discontinued (if applicable). Patients were required to have VAS actual pain intensity at baseline of ≥50 mm for the most severely affected (target) knee joint after activity. (The pain requirement at baseline following washout [≥50 mm] was greater than at screening [≥40 mm]; thus, an increase in pain from screening to baseline was required for study entry.) Patients were subsequently randomly assigned (in the ratio 2:2:1) to oral treatment with lumiracoxib 400 mg once daily, celecoxib 200 mg twice daily, or placebo. Blinding was maintained by the double-dummy technique. Following 7 days of study medication, patients had a final clinic visit, during which final pain assessments were made, pain records from the patient diary were reviewed, and repeat functional status and safety assessments were made. Each patient was contacted by telephone for follow up approximately 14 days after study completion.

Compliance was evaluated by quantifying the returned study medication. Patients taking at least 80% of prescribed daily medication were deemed compliant.

### Efficacy assessments

In the single-dose pain assessment phase of the study, the analgesic efficacy of a single dose of study medication was evaluated. On the first day of treatment each patient rated actual pain intensity (100 mm VAS, after activity) for the target joint immediately before and at 3 and 5 hours after the first dose of study medication. The primary efficacy variable was pain intensity difference (PID) between the baseline pain assessment and the mean of the 3-hour and 5-hour pain assessments on the first day of treatment.

Secondary variables included evaluation of treatment over 7 days during the multiple-dose pain assessment phase. Patients rated actual pain intensity, average pain intensity and worst pain intensity using a 100 mm VAS after activity in the morning and evening immediately before taking the next dose of study medication. Actual pain intensity difference (APID), average pain intensity difference (AVPID) and worst pain intensity difference (WPID) from baseline were calculated. Pain relief was recorded each evening (between 18:00 hours and 22:00 hours) and morning (30–60 minutes after getting up) after taking the study medication using a 5-point categorical scale (0 = none, 1 = little, 2 = moderate, 3 = a lot, 4 = complete).

In order to evaluate the effect of study medication on usage of rescue medication, all patients were requested to record the date and time of rescue acetaminophen use after randomization.

Functional status was assessed at baseline and study completion using the total and all three subscales of Western Ontario and McMaster Universities Osteoarthritis Index (WOMAC™) LK3.1 questionnaire (Likert scale) [24]: pain; stiffness; and difficulty in performing daily activities (DPDA).

At study end (or premature discontinuation), all patients completed a global evaluation of treatment effect, which was recorded on a 4-point categorical scale (1 = poor, 2 = fair, 3 = good, 4 = excellent).

### Safety and tolerability assessments

All adverse events reported by the patient or discovered by the investigator during the study period were recorded and evaluated in terms of seriousness, severity and potential relationship to study medication. Safety assessments consisted of routine laboratory tests (haematology, biochemistry and urinalysis), measurement of vital signs and 12-lead electrocardiogram recordings, which were completed at screening/baseline and study end.

### Statistical analysis

The primary efficacy variable was the APID between the baseline pain assessment and the mean of the 3-hour and 5-hour actual pain assessments after the first dose of study medication.

To maintain an overall 5% significance level, a closed-test procedure with a predefined sort order of the three hypothesis tests (each at the 5% two-sided level) of the primary variable was used [25]: superiority of lumiracoxib 400 mg once daily versus placebo; noninferiority of lumiracoxib 400 mg once daily versus celecoxib 200 mg twice daily; and superiority of lumiracoxib 400 mg once daily versus celecoxib 200 mg twice daily. In addition, in a prespecified, exploratory manner, celecoxib was assessed versus placebo to examine the sensitivity of the trial.

Use of sample sizes of 132 (lumiracoxib 400 mg once daily), 132 (celecoxib 200 mg twice daily) and 66 (placebo group) resulted in powers of 94%, 98% and 80%, respectively, to reject the three hypotheses in turn. The noninferiority margin of lumiracoxib 400 mg once daily to celecoxib 200 mg twice daily was predefined as 5 mm. An overall dropout rate of 10% and a common standard deviation of 30 mm were assumed.

Analysis of efficacy was performed for all randomized patients who had been exposed to study medication (intent-to-treat population). All randomized patients were included in the safety analysis.

PID was analysed using analysis of covariance (ANCOVA). The statistical fixed-effects model considered treatment and centre as main effects, whereas baseline actual pain was used as a covariate. Data from small centres were pooled using a predefined algorithm.

APID, AVPID and WPID were analyzed at each time point relative to baseline, using ANCOVA (as described above), and pair-wise comparisons of treatment groups were calculated. Pain relief was analyzed using a multiple logistic regression model with treatment as an explanatory variable. The WOMAC™ Total and subscale scores were analyzed using an ANCOVA model, fitting scores at baseline, pooled centre and treatment group. Finally, pair-wise comparisons of treatment groups for the patient's global evaluation of treatment effect were performed using a Cochran–Mantel–Haenszel test, adjusting for pooled centre. All secondary variables were analyzed on the intent-to-treat population and all tests were two sided, using a 5% level of significance.

## Results

After screening 418 patients, a total of 364 were enrolled into the study and received at least one dose of study medication (lumiracoxib 400 mg once daily [*n *= 144], celecoxib 200 mg twice daily [*n *= 145] and placebo [*n *= 75]). Five patients (1.4%) withdrew from the study, four from the lumiracoxib group (three due to adverse events and one for unsatisfactory therapeutic effect) and one from the celecoxib group (due to protocol violation).

Overall, the treatment groups were balanced for baseline demographics and clinical characteristics (Table 1). Between group differences in the distribution of female patients and patients aged ≥75 years were not statistically significant (sex, *P *= 0.3105; age, *P *= 0.8416) and were not considered to have influenced the outcome of the study.

More than 90% of patients in each treatment group achieved satisfactory compliance with prescribed medication during the course of the study.

### Efficacy

#### Single-dose pain assessment phase

The three main hypotheses of the primary efficacy variable (APID between the baseline pain assessment and the mean of the 3-hour and 5-hour actual pain assessments after the first dose of study medication) were tested in sequence. First, lumiracoxib exhibited a statistically superior analgesic effect to that of placebo, with an estimated treatment–placebo difference (least square mean) of 5.8 mm (95% confidence interval [CI] 1.89–9.75 mm; *P *= 0.004; Table 2). Second, lumiracoxib was confirmed to be noninferior to celecoxib in this analysis, because the lower limit of the 95% CI for the difference between lumiracoxib and celecoxib (lumiracoxib–celecoxib difference) was less than -5 mm. In the third hypothesis test, lumiracoxib was not significantly superior to celecoxib in terms of the analgesic efficacy of the first dose (estimated least square mean difference in favour of lumiracoxib: 2.2 mm, 95% CI -1.06 to +5.45 mm; *P *= 0.185). Furthermore, celecoxib did not have a statistically superior analgesic effect compared with placebo (estimated treatment–placebo difference [least square mean]: 3.6 mm, 95% CI -0.29 to +7.54 mm; *P *= 0.069; Table 2).

As early as 3 hours after the dose on the first day, lumiracoxib exhibited superior analgesic efficacy to that of placebo (estimated treatment–placebo difference: 4.5 mm, 95% CI 0.44–8.63 mm; *P *= 0.03). At the same time point, the percentages of patients who assessed their pain relief as either 'a lot' or 'complete' relative to baseline were 12.5%, 5.5% and 9.3% in the lumiracoxib, celecoxib and placebo groups, respectively. Corresponding values 5 hours after the dose were 11.1%, 10.3% and 8.0%, respectively.

#### Multiple-dose pain assessment phase

**Figure 1:**
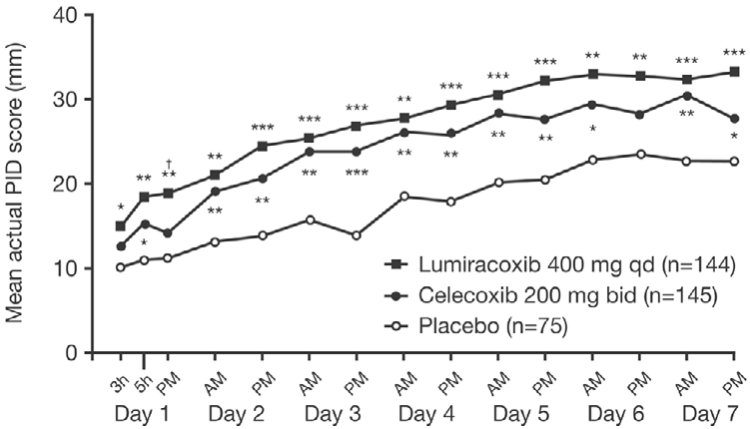
Mean actual pain intensity difference during seven days of treatment.

For the second assessment phase (multiple dosing over 7 days), lumiracoxib was statistically superior to placebo at all time points and celecoxib was superior to placebo at most time points (Figure 1). Lumiracoxib was numerically superior to celecoxib throughout the study, but this achieved statistical significance only in the evening assessment of the first day of treatment (*P *= 0.022). Although not tested statistically, celecoxib-treated patients appeared to have somewhat less pain relief at the evening assessments compared with morning assessments. At study end, the estimated differences for APID relative to placebo were statistically significant for both lumiracoxib (10.7 mm, 95% CI 4.70–16.7 mm; *P *= 0.001) and celecoxib (8.7 mm, 95% CI 2.68–14.66 mm; *P *= 0.005). However, no significant difference was apparent between the active treatment groups at study end (estimated difference for lumiracoxib versus celecoxib in APID: 2.0 mm, 95% CI -2.94 to +7.01 mm; *P *= 0.421).

At study end, 13.9% of patients receiving lumiracoxib experienced 'complete' pain relief at study end compared with 5.5% of patients receiving celecoxib and 5.3% of patients receiving placebo. Furthermore, the percentages of patients who assessed their pain relief as 'a lot' were 34.7%, 30.3% and 22.7% in the lumiracoxib, celecoxib and placebo groups, respectively. With respect to pain relief, lumiracoxib was significantly superior to placebo (*P *= 0.001) whereas celecoxib just failed to achieve significance (*P *= 0.051). There was no significant difference between the active treatment groups at study end (*P *= 0.076).

The majority of patients (87% of lumiracoxib-treated patients, 82% of celecoxib-treated patients and 84% of placebo-treated patients) did not require rescue acetaminophen during the study.

Overall, both active treatment groups were characterized by significantly improved WOMAC™ total scores compared with placebo at study end (Table 3). In addition, lumiracoxib was significantly superior to celecoxib (*P *= 0.026) with regard to the WOMAC™ total score. Lumiracoxib was superior to both celecoxib (*P *= 0.015) and placebo (*P *< 0.001) with regard to the DPDA subscale score, with the difference between the celecoxib and placebo groups just failing to achieve significance (*P *= 0.056). Mean changes from baseline in WOMAC™ total and subscale scores are also shown in Table 3.

**Figure 2:**
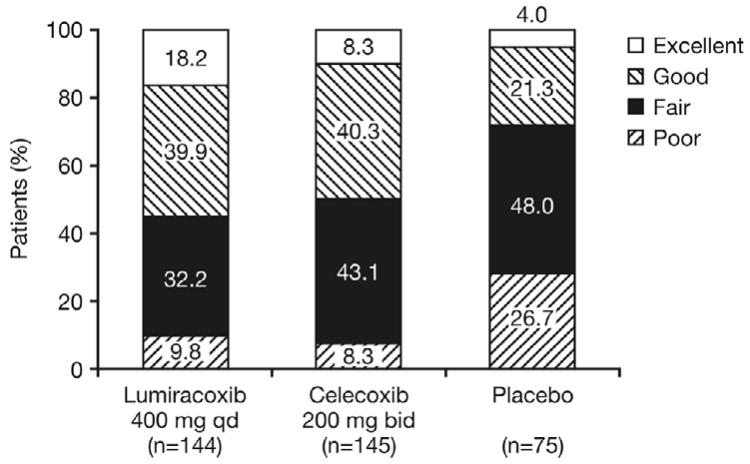
Patient's global evaluation of treatment effect at study end.

In terms of patient's global evaluation of treatment effect, substantially more patients in the lumiracoxib group (58.1%) assessed treatment effect as either 'excellent' or 'good' at study end compared with 48.6% of celecoxib-treated patients and 25.3% of placebo-treated patients (Figure 2). For both active treatments, a highly significant difference relative to placebo was apparent (lumiracoxib, *P *< 0.001; celecoxib, *P *= 0.001); however, there was no difference between active treatment groups (*P *= 0.100).

### Safety and tolerability

Only one serious adverse event was reported (silent myocardial infarction in a celecoxib-treated patient). This event was suspected to be related to the study drug but it did not lead to premature discontinuation because it was detected on electrocardiogram recordings on the final study day. Three patients (2.1%) in the lumiracoxib group were prematurely withdrawn from the study because of adverse events, which included moderate bronchitis and mild nasopharyngitis in one patient each (both were considered to be unrelated to study drug). A further patient was withdrawn because of severe upper abdominal pain that was considered by the investigator to be related to study drug. Of note, this patient had previously (six months before study entry) experienced an episode of abdominal pain during short-term therapy with celecoxib.

Overall, a comparable and low proportion of patients in each treatment group reported at least one adverse event during the study: 14.6% in the lumiracoxib group, 11.0% in the celecoxib group, and 13.3% in the placebo group. Gastrointestinal disorders were the most frequent adverse events, occurring with greater frequency in both active treatment groups than with placebo. The majority of adverse events in each treatment group were of mild severity. Moderate adverse events were infrequent and were not associated with a specific body system.

Adverse events assessed by the investigator as being possibly study drug related occurred in 6.3%, 6.2% and 8.0% of patients in the lumiracoxib, celecoxib and placebo groups, respectively. Overall, adverse events affecting the gastrointestinal system were most frequently reported as being possibly study drug related (4.2%, 4.8% and 2.7% of patients in the lumiracoxib, celecoxib and placebo groups, respectively). There were no relevant changes in vital signs or electrocardiographic variables between baseline and study end in any of the treatment groups.

## Discussion

This short-term study established that lumiracoxib, a novel COX-2 selective inhibitor, provides rapid and effective analgesia in patients with OA of the knee. The primary efficacy variable (mean PID 3–5 hours after dose) was similar to those used in other single-dose studies of COX-2 selective inhibitors in acute pain [26] or in patients with OA flare [27]. Celecoxib was chosen as the active comparator because of its established analgesic efficacy in patients with OA of the knee [28,29].

Previous studies have shown that lumiracoxib provides significant pain relief over 13 weeks of treatment in patients with OA compared with placebo [30,31]. In the present study we found that the first dose of lumiracoxib 400 mg decreased OA pain intensity (100 mm VAS) 3–5 hours after dose by 19.8 mm (*P *= 0.004, versus placebo) compared with a mean decrease of 16.8 mm (*P *= 0.069, versus placebo) with celecoxib 200 mg and 13.4 mm with placebo. Lumiracoxib was noninferior but not significantly superior to celecoxib in this analysis 3–5 hours after dose. These findings are consistent with earlier pharmacodynamic studies with lumiracoxib, which found the difference from baseline in mean VAS scores at 4 hours after dosing to be >20 mm with lumiracoxib 400 mg compared with <5 mm in placebo-treated patients [32]. The results reported here are similar to findings with other COX-2 selective inhibitors, where the speed of onset of analgesia was studied in an OA population meeting OA flare criteria [27]. At 3 hours after the dose, the mean decrease in OA pain intensity (100 mm VAS) was 21.7 mm with valdecoxib 10 mg and 19.8 mm with rofecoxib 25 mg, as compared with 15.5 mm with placebo (*P *< 0.01). In addition, it has been reported that a 30–33% decrease in VAS pain scores represents a clinically meaningful change in either acute or chronic pain [33,34]. For lumiracoxib 400 mg, a 30% decrease from actual pain at baseline was observed at the 3–5 hour post-dose assessment, suggesting that this change in pain intensity was clinically relevant.

The primary objective of the study was to examine the single-dose onset of analgesia with lumiracoxib. As such, a dose of lumiracoxib appropriate for acute use (400 mg) was used. Previous studies with this dose of lumiracoxib in acute pain have shown that it provides rapid and effective analgesia [35-37]. The recommended chronic dose of lumiracoxib is 100 mg daily. In order for the active comparison to be relevant, a dose of celecoxib considered to be appropriate for acute pain was chosen (200 mg twice daily) [22]. (The recommended dose of celecoxib in OA is 200 mg daily, administered as a single dose or as 100 mg twice daily [22].)

Both lumiracoxib and celecoxib had statistically significant efficacy compared with placebo in terms of secondary efficacy variables assessed during the multiple-dose assessment period, including APID, AVPID, WPID and extent of pain relief. The significant treatment–placebo differences in actual pain intensity (100 mm VAS) at study end (10.7 mm for lumiracoxib 400 mg once daily, *P *= 0.001; 8.7 mm for celecoxib 200 mg twice daily, *P *= 0.005) were comparable with those previously reported after 13 weeks of treatment (8.8 mm for lumiracoxib 400 mg once daily; 6.3 mm for celecoxib 200 mg once daily) [31]. At study end, no significant differences were observed between active treatments in terms of APID. Substantially more patients in the lumiracoxib group assessed treatment effect as 'excellent' or 'good' at study end compared with those who received celecoxib (58% and 49%, respectively). Interestingly, only around 25% of placebo recipients rated treatment as 'poor' at the end of the study, despite a progressive increase in APID during treatment. This 'placebo effect' is not unique to the treatment of patients with joint disorders [38] and is perhaps attributable to psychological mechanisms such as an awareness of being closely observed and active compliance with the presumed wishes of researchers. It is notable, however, that against this background lumiracoxib maintained significant superiority in terms of analgesic efficacy.

Patients received celecoxib 200 mg in the morning and evening, whereas lumiracoxib was given in the morning only, with a lumiracoxib-matched placebo administered in the evening. The multiple-dose actual PID scores for lumiracoxib over the 7-day treatment period show that the analgesic effect of lumiracoxib is maintained over a 24-hour period and therefore supports once-daily administration.

Evaluation of WOMAC™ subscales in the study allowed assessment of the effect of treatment on aspects of patients' daily lives relating to their condition, including pain, stiffness and the ability to perform daily activities. Overall, both lumiracoxib and celecoxib were associated with significantly lower pain and stiffness WOMAC™ subscale scores than placebo at study end. This is of particular significance for patients with knee OA, whose mobility and quality of life can be severely impaired [39]. Treatment–placebo differences in the WOMAC™ total score at 7 days (8.9 for lumiracoxib 400 mg once daily; 4.8 for celecoxib 200 mg twice daily) were comparable with those seen in longer 13-week studies with lumiracoxib and celecoxib (7.5 for lumiracoxib 400 mg once daily; 6.0 for celecoxib 200 mg once daily) [31]. It has been suggested that the minimal difference perceived (MDP) clinically from baseline in WOMAC™ DPDA score should be the minimal difference that is perceived by 75% of patients (MDP75), and a recent study of 1354 patients reported the MDP75 for the WOMAC™ DPDA subscale to be 5.2 [40]. In this study, the change from baseline in the DPDA subscale score was 15.4 for lumiracoxib 400 mg once daily compared with 12.3 for celecoxib 200 mg twice daily. This suggests that lumiracoxib provided clinically meaningful improvements in the ability of patients to perform daily activities during this 7-day study.

Lumiracoxib was well tolerated in this short study. The incidence of adverse events was comparable between all three groups. Previous studies of lumiracoxib tolerability over 13 weeks of treatment have found that lumiracoxib 200 mg or 400 mg daily has gastrointestinal tolerability that is superior to that of traditional NSAIDs including diclofenac, ibuprofen and naproxen [41].

## Conclusion

Lumiracoxib 400 mg (four times the recommended chronic dose in OA) had a rapid onset of action in patients with OA of the knee, with an analgesic effect significantly superior to that of placebo that was demonstrated as early as three hours following the first dose. In addition, analgesia was maintained throughout the dosing interval; lumiracoxib was superior to placebo for overall pain relief at both morning and evening assessments throughout the study. The rapid onset of analgesia represents a useful attribute in the management of patients with OA, who are likely to require intermittent *pro re nata *medication.

## Abbreviations

ANCOVA = analysis of covariance; APID = actual pain intensity difference; AVPID = average pain intensity difference; CI = confidence interval; COX = cyclo-oxygenase; DPDA = difficulty in performing daily activities; ITT = intent to treat; MDP = minimal difference perceived; NSAID = nonsteroidal anti-inflammatory drug; OA = osteoarthritis; PID = pain intensity difference; SD = standard deviation; VAS = visual–analogue scale; WOMAC™ = Western Ontario and McMaster Osteoarthritis Index; WPID = worst pain intensity difference.

## Competing interests

Taiwo OA Fashola, Helen J Thurston, Klaus J Burger and Ulrich Trechsel were employees of Novartis when the study was conducted. All other authors have declared that they have no competing interests.

## Authors' contributions

RHW, ES, GK, RM, HR and PS were investigators in the study, TOAF was the clinical trial leader (participating in study design and coordination), HJT performed the statistical analysis, KJB was Medical Advisor for Germany (participating in study design and coordination) and UT was the pain clinical programme leader (participating in study design and coordination). All authors were involved in data interpretation and participated in drafting the manuscript, and read and approved the final version.

## Figures and Tables

**Table 1:** Patient demographics and baseline disease characteristics

Parameter/characteristic	Lumiracoxib 400 mg once daily (*n *= 144)	Celecoxib 200 mg twice daily (*n *= 145)	Placebo (*n *= 75)
Age (years; mean ± SD)	64.7 ± 7.8	65.3 ± 9.5	64.8 ± 9.0
Males (*n *[%])	66 (45.8)	54 (37.2)	33 (44.0)
Females (*n *[%])	78 (54.2)	91 (62.8)	42 (56.0)
Disease duration (years; mean ± SD)	7.3 ± 6.7	7.5 ± 6.7	7.6 ± 8.6
VAS actual^a ^pain intensity (mm; mean ± SD)	65.8 ± 11.5	64.1 ± 11.5	64.4 ± 12.4
VAS worst^b ^pain intensity (mm; mean ± SD)	75.7 ± 11.5	75.5 ± 11.8	74.3 ± 11.2
VAS average^c ^pain intensity (mm; mean ± SD)	57.9 ± 12.5	56.9 ± 11.6	57.0 ± 14.8
WOMAC™ scores (mean ± SD)			
Total	52.3 ± 12.6	53.9 ± 12.5	53.2 ± 11.6
Pain	10.6 ± 2.9	11.1 ± 2.9	10.8 ± 2.6
Stiffness	4.0 ± 1.7	4.0 ± 1.7	4.1 ± 1.3
DPDA	37.8 ± 9.2	38.8 ± 9.2	38.3 ± 8.7

^a^VAS actual pain intensity = mean of 3-hour and 5-hour assessments. ^b^VAS worst pain intensity = worst pain intensity in the past 12 hours. ^c^VAS average pain intensity = average pain intensity in the past 12 hours. DPDA, difficulty in performing daily activities; SD, standard deviation; VAS, visual–analogue scale; WOMAC™, Western Ontario and McMaster Universities Osteoarthritis Index.

**Table 2:** Mean actual PID for 3-hour and 5-hour time points after first dose

Mean actual PID (10 mm VAS)	Lumiracoxib 400 mg (*n *= 144)	Celecoxib 200 mg (*n *= 145)	Placebo (*n *= 75)
Mean ± SD	19.8 ± 16.5**	16.8 ± 15.9^†^	13.4 ± 12.9
Median (range)	16.0 (-15.5, 74.5)	15.5 (-31.0, 58.5)	12.5 (-9.5, 48.5)

**P = 0.004, pair-wise comparison versus placebo using least square means; ^†^*P *= 0.069, pair-wise comparison versus placebo using least square means. PID, pain intensity difference; SD, standard deviation; VAS, visual–analogue scale.

**Table 3:** Change from baseline and treatment differences in WOMAC™ total and subscale scores at study end

	WOMAC™ total score	WOMAC™ pain subscale score	WOMAC™ DPDA subscale score^a^	WOMAC™ stiffness subscale score
Change from baseline at study end (mean ± SD)				
Lumiracoxib 400 mg daily (*n *= 144)	-21.3 ± 19.9	-4.4 ± 4.4	-15.4 ± 14.6	-1.5 ± 1.9
Celecoxib 200 mg twice daily (*n *= 145)	-17.6 ± 14.2	-4.0 ± 3.3	-12.3 ± 10.5	-1.3 ± 1.8
Placebo (*n *= 75)	-12.5 ± 13.4	-2.7 ± 3.2	-9.0 ± 9.8	-0.8 ± 1.5
Estimated treatment differences at study end (least square means [95% CI])				
Lumiracoxib versus placebo	-8.9 (-13.30 to -4.56)***	-1.8 (-2.77 to -0.83)***	-6.4 (-9.62 to -3.23)***	-0.7 (-1.14 to -0.31)***
Celecoxib versus placebo	-4.8 (-9.15 to -0.44)*	-1.1 (-2.02 to -0.10)*	-3.1(-6.30 to +0.07)	-0.6 (-1.00 to -0.18)**
Lumiracoxib versus celecoxib	-4.1 (-7.76 to -0.51)*	-0.7 (-1.54 to +0.06)	-3.3 (-5.96 to -0.66)*	-0.1 (-0.47 to +0.21)

^a^Data missing for one patient in each of the lumiracoxib and celecoxib treatment groups. *P < 0.05, **P < 0.01, ***P = 0.001. CI, confidence interval; DPDA, difficulty in performing daily activities; SD, standard deviation; WOMAC™ = Western Ontario and McMaster Universities Osteoarthritis Index.
